# Gene Expression of Hormone Receptors and Growth Factors in Intact and Neutralized Female Dogs, Both Healthy and with Cutaneous Mast Cell Tumors

**DOI:** 10.3390/ani16091364

**Published:** 2026-04-29

**Authors:** Florencia Sollier, Victoria de Brun, Daniela Izquierdo, Ana Meikle

**Affiliations:** 1 Small Animal Clinic and Surgery Unit, Faculty of Veterinary Medicine, University of the Republic, Route 8 km 18, Montevideo 13000, Uruguay; dizquierdo.caquias@gmail.com; 2 Laboratory of Animal Endocrinology and Metabolism, Faculty of Veterinary Medicine, University of the Republic, Route 8 km 18, Montevideo 13000, Uruguay; videbrun@gmail.com (V.d.B.); meikleana@gmail.com (A.M.)

**Keywords:** gonadectomy, dog, cancer, gonadotropin receptor

## Abstract

Mast cell tumors are the most common skin neoplasm in dogs and may be influenced by reproductive status. A gonadectomy modifies gonadotropin levels and may affect the expression of hormonal receptors and proliferative factors; however, the evidence for this remains limited. The objective of this study was to evaluate the expression of *VEGF*, *IGF-1*, *PCNA*, *Ki-67*, *c-KIT*, *LHR*, *FSHR*, and *ERα* in intact and neutralized female dogs with and without MCTs. Gene expression in the skin samples was quantified by a real-time PCR, and four groups were included: intact controls (*n* = 10), neutralized controls (*n* = 10), intact MCT (*n* = 9), and neutralized MCT (*n* = 10). Tumor presence was associated with increased expression of *LH* and *FSH* receptors and *c-KIT*, while angiogenic and proliferative factors (*PCNA*, *Ki-67*, *IGF-1*, *VEGF*, and *ERα*) showed lower expression. In dogs with MCTs, a gonadectomy was associated with higher *c-KIT* and *VEGF* expression but lower *LHR* mRNA levels. *PCNA* expression was lower in the neutralized MCT dogs compared to the neutralized controls, whereas no differences were observed in the intact dogs. Additionally, *ERα* expression was higher in the neutralized control dogs than in the intact controls, with no differences detected in the MCT dogs. These findings suggest that reproductive status is associated with differential regulation of molecular pathways involved in canine MCT biology.

## Simple Summary

Mast cell tumors are the most common skin cancer in dogs, and previous studies have suggested that its development may be influenced by reproductive status and the hormonal changes associated with spaying. However, the evidence for this relationship remains limited. The aim of this study was to assess whether reproductive status is associated with changes in the biological processes related to tumor development in female dogs with and without mast cell tumors. Skin samples from intact and neutralized female dogs, both healthy and affected by a tumor, were analyzed to evaluate the activity of genes involved in cell growth, hormone signaling, and tumor behavior. The results show that the presence of a tumor was associated with increased activity of genes related to hormone receptors and tumor progression, while the genes associated with cell growth and tissue regulation showed lower activity. In dogs with mast cell tumors, spaying was linked to additional changes in gene activity compared to intact dogs. Overall, these findings suggest that reproductive status may influence the key biological pathways involved in mast cell tumor development in dogs. This knowledge may help improve understanding of the disease and support better clinical decision-making in veterinary practice.

## 1. Introduction

Recent evidence has challenged the concept of gonadectomy as a universally beneficial intervention, highlighting breed-, sex-, and age-dependent effects on the risk of non-reproductive diseases, including cancer. In this context, reproductive status and its consequent hormonal alterations have been suggested to influence the presentation of mast cell tumors (MCTs), although this association remains controversial [1,2,3]. In gonadectomized mammals, there is no negative feedback from gonadal steroids, resulting in supra-physiological circulating gonadotropin concentrations [4]. Gonadotropin receptors have been found in healthy skin [5], and it has been proposed that their increased expression may play an important role in the pathogenesis of various conditions associated with gonadectomy, such as cutaneous neoplasms [4,5]. Indeed, Li et al. reported that the expression of LHR and FSHR in canine mammary tumors was associated with dysregulation in tumor malignancy and reproductive status [6]. As far as we know, there is only one study that has reported in MCT cases increased immunopositively cells to LHR in neutralized females when compared to intact males and females [7].

It is known that estrogen-sensitive breast cancer cells display an elevated expression of insulin-like growth factor (IGF-1) when compared to normal epithelial cells [8]. In these tumors, ERα is a key therapeutic target due to the synergistic crosstalk between IGF-1R and ERα, resulting in enhanced activation of both receptors’ signaling cascades [9]. While this interaction pathway is well characterized in breast cancer, its potential role in MCTs remains unexplored. IGF-1 expression is associated with tumor pathogenesis due to the mitogenic and anti-apoptotic properties of its corresponding receptors [10]. The experimental evidence suggests that IGF-1 significantly influences the transformation, infiltrative growth, and metastasis of tumor cells [11]. However, there is no evidence regarding its relationship with the pathogenesis of MCTs. Many other factors modulate angiogenesis, which is essential for the development and metastasis of solid tumors because tumor-associated vessels can supply oxygen and nutrition to tumor cells, and they have been investigated as tumor prognostic factors [12], such as vascular endothelial growth factor (VEGF). As far as we know, there is only one study that shows that canine MCT cells express VEGF and its receptors, and the authors suggest that VEGF is not an autocrine growth regulator [13].

Estimating the tumor proliferation potential is useful for determining its malignancy. Ki-67 and proliferating cell nuclear antigen (PCNA) are biomarkers of proliferation: high Ki-67 expression in MCTs is associated with increased mortality, local recurrence rates, and metastasis, independent of the histological grade [14]. Moreover, it is known that mutations in the c-KIT proto-oncogene are associated with the pathogenesis and aggressiveness of MCTs; however, not all MCTs exhibit c-KIT mutations [13]. On the other hand, even if c-KIT mRNA levels in canine MCT tissues are analyzed using quantitative PCR (qPCR), information about its overall expression levels in MCTs according to the reproductive state remains limited [15,16].

Thus, we hypothesized that the expression of proliferative factors and hormone receptors is affected by the presence of MCTs and that this differential expression is modulated by the reproductive state. The objective of our study was to investigate the presence and the gene of expression of VEGF, IGF-1, PCNA, Ki-67, c-KIT, LHR, FSHR, and ERα in neutralized and intact dogs with or without MCTs.

## 2. Materials and Methods

The study was approved by the Ethics Committee of the Faculty of Veterinary Medicine, University of the Republic, Uruguay (CEUA approval number: 111900-000382-21). This study did not involve the use of cell lines; all data were obtained from client-owned animals. In all cases, informed consent was obtained from the owners prior to participation. Tissue samples were collected at the Small Animal Surgery and Anesthesia Unit and subsequently processed at the Laboratory of Nuclear Techniques.

### 2.1. Animals and Tissue Samples

Female dogs diagnosed with MCTs through a cytological examination and with a surgical indication, admitted to the Veterinary Hospital Center between the years 2023 and 2024, were included in the study. Tumor staging was performed according to the criteria described by Horta et al. (2018) [17], and only dogs presenting with a single cutaneous mass without evidence of regional lymph node or distant metastasis were included. Before the surgical procedure, a complete blood count, renal profile, hepatic profile, coagulation profile, and urine analysis were conducted, along with thoracic radiographs and abdominal ultrasound. On the day of the procedure, the animals were premedicated with methadone (0.4 mg/kg) and acepromazine (0.03 mg/kg) via intramuscular injection, induced with intravenous propofol (5 mg/kg), and maintained under general anesthesia with isoflurane at 2% in a closed-circuit inhalation system. Antibiotic therapy was administered 30 min before surgery with amoxicillin (22 mg/kg) intramuscularly. For analgesia, meloxicam (0.2 mg/kg) was given intravenously, and fentanyl was administered as needed. Tumor excision was performed based on the most recent Brazilian consensus on MCTs [18]. Tissue sampling was performed with caution to exclude necrotic areas; for large tumors, representative material was obtained from the peripheral region, avoiding the central portion. A sample of neoplastic tissue was immediately placed in liquid nitrogen and subsequently stored at −80 °C until transcript analysis. The control group consisted of healthy females, confirmed through a complete blood count, renal and hepatic function, coagulation profile, and urine analysis. The neutralized females included in the control group underwent an elective ovariohysterectomy, and samples were obtained during routine sterilization procedures. The intact control dogs were presented for non-oncologic conditions, mainly traumatic or orthopedic disorders requiring surgical intervention. Animals with any history or clinical evidence of neoplastic, dermatologic, or metabolic diseases were excluded from the control group to avoid potential bias related to inflammatory or neoplastic processes that could affect mast cell distribution. Therefore, no samples from dogs undergoing surgery for other tumor types were included. For this procedure, the same anesthetic protocol used for the MCT patients was applied, and during the gonadectomy, skin biopsies were obtained using a 6 mm punch from the lateral trunk region. These samples were also stored at −80 °C until transcript determination.

Following histopathological confirmation, 19 female dogs with cutaneous MCTs were selected based on their reproductive status and tumor grade, in order to establish homogeneous groups. Regarding their gonadal status, the neutralized females were considered neutered only when their ovariohysterectomy had been performed at least one year before inclusion in the study, in order to minimize possible residual hormonal effects. Fisher’s exact test was applied to evaluate the association between tumor grade and gonadal status, yielding a test statistic of 1 and indicating no significant association (*p* > 0.05). Therefore, the four experimental groups were: control intact (*n* = 10), control neutralized (*n* = 10), MCT intact (*n* = 9) and MCT neutralized (*n* = 10). The mean age of dogs in the MCT group was 7.92 ± 2.72 years and in the control group it was 6.05 ± 4.20 years, with no significant difference between groups (*p* > 0.05). The information regarding the individual breeds, ages in each group and reproductive status is included in Supplementary Table S1.

### 2.2. Histopathological Classification

**Figure 1:**
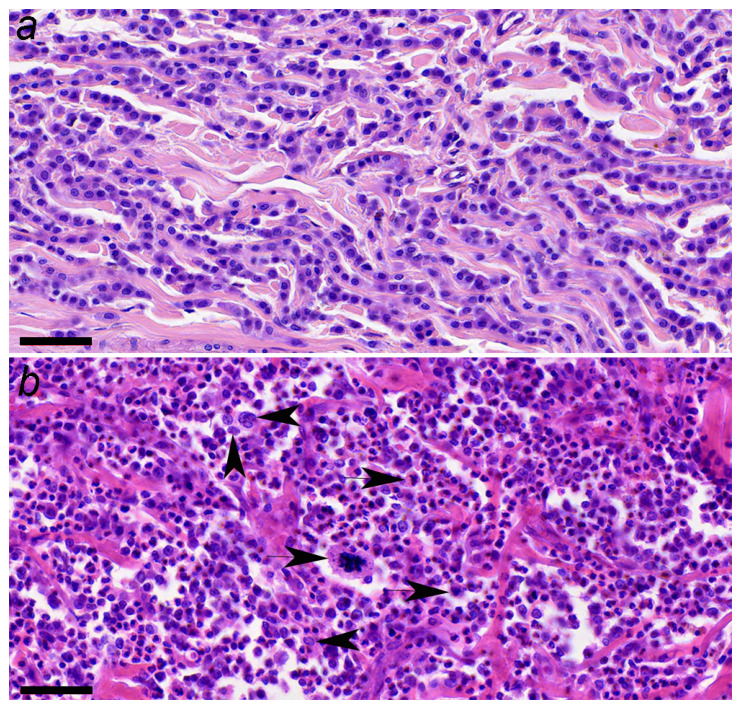
(**a**) Canine low-grade mast cell tumor, showing a regular pattern of proliferation of well-differentiated tumoral mast cells. High power field (HPF); HE (40×); scale bar = 50 µm. (**b**) Canine high-grade mast cell tumor, showing an extremely irregular pattern of proliferation of anaplastic tumoral mast cells, with three mitotic figures (arrows) and three mast cells with double or multiple nuclei in the same HPF (arrowheads). HE (40×); scale bar = 50 µm.

All samples were evaluated at the Pathology Unit of the Faculty of Veterinary Medicine, and only those classified as grade 2 according to Patnaik, and as either low- or high-grade according to Kiupel et al. [19], were included to minimize the variability related to tumor grading (Figure 1). The mitotic index (MI) was assessed on hematoxylin–eosin (HE)-stained sections by three pathologists. The number of mitotic figures in 12 high-power fields (HPFs, 400×) were counted, corresponding to an area of 2.37 mm^2^, using an optical microscope (FN 20; Nikon Eclipse E200, Nikon, Tokyo, Japan). MI data were available for 11 of 19 tumors (57.9%). Among these, 54.5% (6/11) exhibited a low MI (<5 mitoses per 12 HPFs), while 45.5% (5/11) showed a high MI (≥5 mitoses per 12 HPFs). The remaining 8 cases lacked MI information due to incomplete histopathological records.

### 2.3. Estradiol-17β and Progesterone Concentrations

Serum concentrations of estradiol-17β and progesterone were measured at the time of sample collection and determined at the Laboratory of Animal Endocrinology and Metabolism, Faculty of Veterinary Medicine. The hormone concentrations were measured by chemiluminescent immunoassay (CLIA) using an Immulite 1000 analyzer (Siemens Healthcare Diagnostics, Los Angeles, CA, USA). The intra-assay coefficient of variation was less than 10%.

### 2.4. RNA Isolation and Reverse Transcription

The Total RNA was extracted from the skin tissue using TRIzol (Invitrogen, Carlsbad, CA, USA) according to the manufacturer’s instructions. The samples were then treated with DNAse using a DNA-Free kit (Invitrogen) to remove genomic DNA contamination. The RNA concentration was determined by spectrophotometry at 260 nm, and the RNA purity was evaluated using a 260/280 absorbance ratio. For each sample, complementary DNA (cDNA) was synthesized by reverse transcription using SuperScript III transcriptase (Invitrogen) with random primers and 500 ng of total RNA as the template.

### 2.5. Quantification of cDNA by Real-Time PCR

The expression of target genes in the skin samples was quantified using qPCR. The analyzed genes included *LHR*, *FSHR*, *ERα*, *PCNA*, *Ki-67*, *c-KIT*, *VEGF*, and *IGF-1*, and the endogenous control was hypoxanthine-guanine phosphoribosyl-transferase (*HPRT*). Their expression was evaluated using gene-specific primers: *LHR* (FWD 5′-AAACCAAAGGCCAGTATTATAACCA-3′ REV 5′-AGTGAAAAAGCCAGCTGCACTAC-3′) (GenBank accession number: AF389885), *FSHR* (FWD 5′-GTGGAGATTTTTCTCTGCAAATG-3′ REV 5′-CAGGAGCAGGGCCATAATT-3′) (GenBank accession number: M65085), *ERα* (FWD 5′-AGGGAAGCTCCTATTTGCTCC-3′ REV 5′-CGGTGGATGTGGTCCTTCTCT-3′) (GenBank accession number: AY033393), *PCNA* (FWD 5′-CGGCTACAACTCCTCTTCG-3′ REV 5′-AGTCCATGCTCTGCAGGTTT-3′) (GenBank accession number:: XM_038433208.1), *Ki-67* (FWD 5′-GGACGGAGAAGCAGAAACAG-3′ REV 5′-ATCCTGTGAGGGTGGTCAAG-3′) (GenBank accession number: XM_038440934.1), *c-KIT* (FWD 5′-CCAGTGTGTGGTTGCAGGAT-3′ REV 5′-CTCAGCTCCTGGACAGAAATACC-3′) (GenBank accession number: NM_001003181.1), *VEGF* (FWD 5′-CTATGGCAGGAGGAGAGCAC-3′ REV 5′-GCAGGATGGCTTGAAGATGT-3′) (GenBank accession number: NM_001003175.2), and *IGF-1* (FWD 5′-CCAGACAGGAATCGTGGATG-3′ REV 5′-GCAGTACATCTCCAGCCTCCTCAGA-3′) (GenBank accession number: NM_001077828). The qPCR reactions were prepared in a final volume of 15 μL containing 7.5 μL SYBRGreen mastermix (Quantimix EASY SYG kit, Biotools B & M Labs, Madrid, Spain), equimolar quantities of the forward and reverse primers (10 mM, Operon Biotechnologies GmbH, Cologne, Germany) and 2 μL of diluted cDNA (1:10 in RNAse/DNAse free water). The samples were analyzed in duplicate in a Rotor-Gene 6000 72-disc rotor (Corbett Life Sciences, Sydney, Australia). The standard amplification conditions were initial denaturation at 95 °C for 5 min, 40 cycles of denaturation at 95 °C for 15 s, annealing at 60 °C for 45 s, and extension at 72 °C for 20 s. The dissociation curves were analyzed at the end of each run to confirm amplicon specificity and exclude primer dimers or DNA contamination. A no-template control (NTC negative control) was included to verify the absence of contaminating DNA. Samples of cDNA from all the dogs were pooled to provide an exogenous control, and five dilutions (100, 50, 25, 12.5, and 6.25 ng/tube) of this pool were used to conduct a linear regression for each gene. The efficiency (E) of the tests was calculated according to the formula E = 10^(−1/slope)^. Gene expression was determined by relative quantification according to the Pfaffl method [20]. A pooled cDNA sample generated from all the dogs was used as an internal calibrator in each run to construct standard curves and determine the amplification efficiency for each target gene. The relative expression values were normalized to the endogenous reference gene HPRT using efficiency-corrected calculations [20]. The expression of HPRT was not affected by the group, reproductive status, or their interaction, confirming its suitability as a stable reference gene for normalization.

## 3. Results

### 3.1. Hormonal Concentrations

The 17β-estradiol concentrations were affected by the gonadal status (*p* = 0.0043), as the intact females had detectable concentrations (15.1 ± 3.4 pg/mL) while the neutralized females had basal concentrations (1.0 ± 2.9 pg/mL). The estradiol concentrations of intact MCT dogs were 22.2 ± 5.0 pg/mL and that of control intact dogs were 8.0 ± 4.7 pg/mL.

The progesterone concentrations were affected by the gonadal status (*p* = 0.0068), as the intact females had detectable concentrations (2.5 ± 1.0 ng/mL) while the neutralized females had basal concentrations (0.17 ± 0.9 ng/mL). The progesterone concentrations of intact MCT dogs were 0.92 ± 1.5 ng/mL and that of control dogs were 4.1 ± 1.4 ng/mL.

### 3.2. Tumor Characteristics and c-KIT mRNA Expression

**Figure 2:**
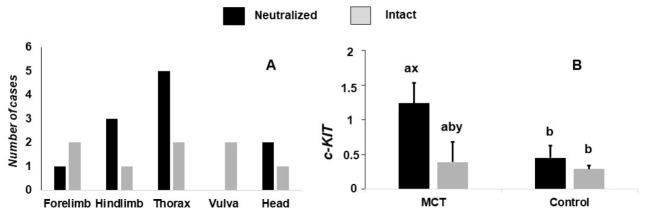
Tumor localization (**A**) and relative expression of c-KIT in neutralized and intact dogs, and healthy (control) dogs and those with mast cell tumors (MCTs). (**B**) Different lowercase letters (a, b) indicate significant differences among groups (*p* ≤ 0.05). Groups sharing at least one letter are not significantly different. Additional letters (x, y) denote comparisons between gonadal status (neutralized vs. intact) within same experimental group.

The proportions of tumor grade, according Kiupel et al., and tumor size were not affected by gonadal status (*p* = 0.827 and *p* = 0.742, respectively). Also, the proportion of tumor localization did not differ in the neutralized and intact dogs (*p* = 0.534). Figure 2A shows the frequency according to the localization. The information regarding the grade, tumor location and tumor size is included in Supplementary Table S2.

The MCT group tended to present with higher *c-KIT* relative expression than the control group (0.82 ± 0.22 vs. 0.29 ± 0.22, *p* = 0.09). The expression of *c-KIT* in the MCT dogs tended to be greater in the neutralized than intact animals (*p* = 0.06). The MCT neutralized dogs presented with greater *c-KIT* expression than the control dogs (*p* = 0.05), but the MCT intact dogs were not different from the control dogs (Figure 2B).

### 3.3. mRNA Expression of Growth and Proliferation Markers

**Figure 3:**
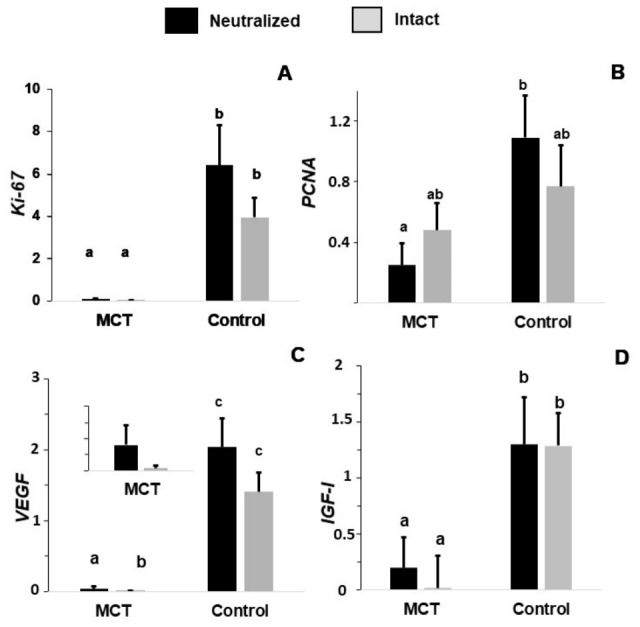
Relative expression of Ki-67 (**A**), PCNA (**B**), VEGF (**C**), and IGF-1 (**D**) in neutralized and intact dogs and in healthy (control) dogs and those with mast cell tumors (MCTs). Different lowercase letters (a, b, c) indicate significant differences among experimental groups (*p* ≤ 0.05). Groups sharing at least one letter are not significantly different. When present, additional letters indicate differences associated with gonadal status within same group.

The expression of *Ki-67* was affected by the group: the MCT group showed basal relative expression compared to the control group (0.07 ± 0.03 vs. 5.18 ± 1.5, *p* < 0.0001) (Figure 3A).

The expression of *PCNA* was affected by the group: the control group had increased *PCNA* expression than the MCT group (0.93 ± 0.14 vs. 0.37 ± 0.14, *p* = 0.0048). The interaction between reproductive status and group (MCT vs. control) showed a trend toward significance (*p* = 0.08), explained by the lower expression found in the MCT neutralized dogs with respect to the neutralized control dogs (*p* < 0.05), which was not found in the intact dogs (Figure 3B).

The expression of *VEGF* was affected by the group (*p* < 0.0001) and the gonadal status (*p* < 0.005). The control group had greater *VEGF* expression than the MCT group (1.72 ± 0.18 vs. 0.02 ± 0.20, *p* < 0.0001). The expression of *VEGF* was significantly higher in the neutralized animals (1.04 ± 0.18 vs. 0.70 ± 0.18, *p* < 0.0001). While no differences were observed in the control animals (intact and neutralized), the MCT neutralized had greater expression than the intact MCT dogs (0.049 ± 0.03 vs. 0.004 ± 0.003, *p* < 0.005) (Figure 3C).

The expression of *IGF-1* was affected by the group (*p* < 0.0001). The expression of *IGF-1* was greater in the control group than in the MCT group (1.3 ± 0.2 vs. 0.1 ± 0.2, *p* < 0.0001) (Figure 3D).

### 3.4. mRNA Expression of Hormonal Receptors

**Figure 4:**
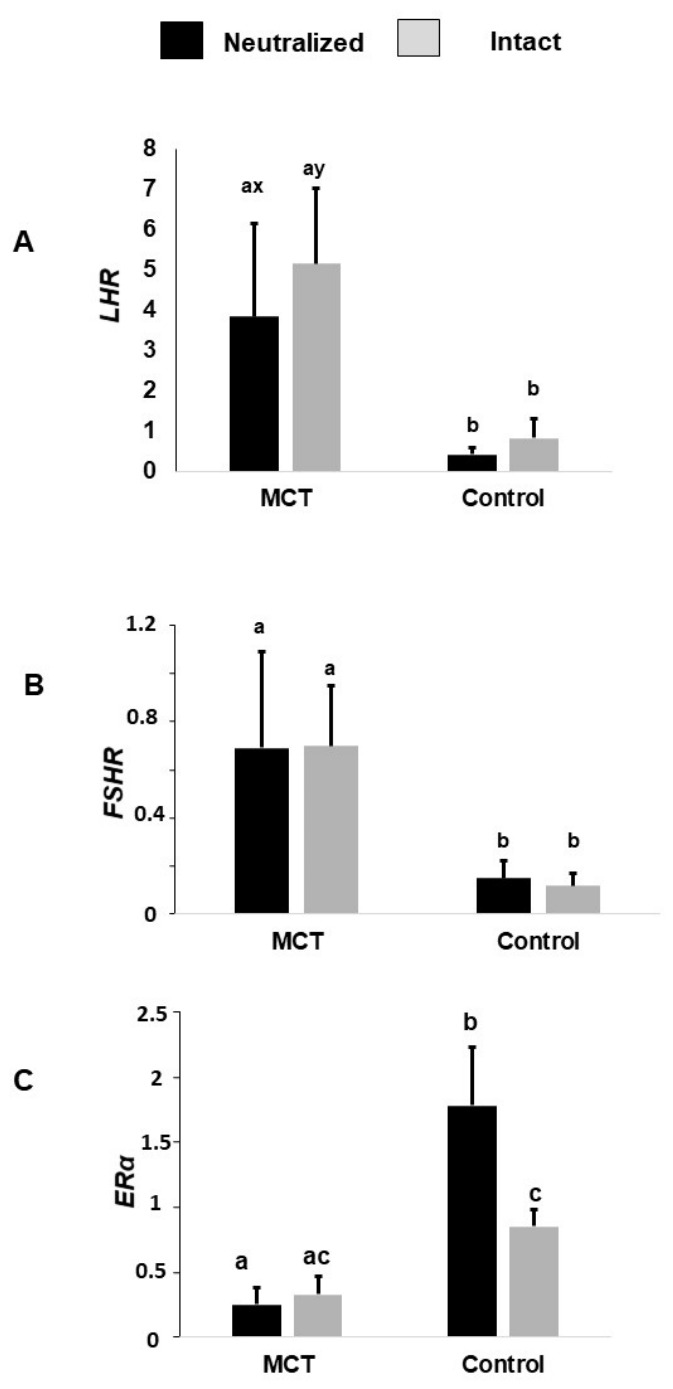
Relative expression of LHR (**A**), FSHR (**B**), and ERα (**C**) in neutralized and intact dogs and healthy (control) dogs and those with mast cell tumors (MCTs). Different lowercase letters (a, b, c) indicate significant differences among experimental groups (*p* ≤ 0.05). Groups sharing at least one letter are not significantly different. Letters x and y indicate differences between gonadal status (neutralized vs. intact) within same group.

The expression of *LHR* was affected by the treatment group, as the MCT group had greater *LHR* expression than the control group (4.5 ± 0.9 vs. 0.6 ± 0.92, *p* = 0.0002). The MCT intact animals tended to show greater expression than the MCT neutralized animals (5.15 ± 1.81 vs. 3.85 ± 2.30, *p* = 0.08) (Figure 4A).

The MCT group had greater *FSHR* expression than the control group (0.69 ± 0.15 vs. 0.13 ± 0.15, *p* = 0.004) (Figure 4B).

The expression of *ERα* was affected by the group (*p* < 0.0001) and the interaction between the group–reproductive status (*p* = 0.08). The control group had increased ERα expression in comparison to the MCT group (1.31 ± 0.19 vs. 0.29 ± 0.18, *p* < 0.0001). While no differences were observed in the MCT animals (intact and neutralized), the control neutralized group presented with increased expression compared to the intact dogs (1.78 ± 0.44 vs. 0.85 ± 0.13, *p* = 0.05) (Figure 4C). The MCT neutralized dogs had lower *ERα* expression than the control neutralized dogs, but no differences were found in the intact dogs.

## 4. Discussion

This study is the first to report the expression of LHR, FSHR and ERα and their association with proliferative factors (VEGF, IGF-1, PCNA, Ki-67, and c-KIT) in neutered and intact dogs with or without canine cutaneous MCTs. The data show differential expression of all transcripts according to health status, while c-KIT, VEGF and LHR expression are associated with gonadal status in the MCT dogs, which raises the possibility that reproductive status may influence the biology of this tumor type. Importantly, however, the present study is based on a quantitative mRNA expression analysis, and it is well recognized that transcriptional levels do not necessarily correlate with protein expression or biological activity. Therefore, the functional significance of the observed differences should be interpreted with caution.

Canine MCT is a biologically and morphologically heterogeneous neoplasm, which complicates the understanding of its biological mechanism. To minimize this heterogeneity, our study included only solitary tumors without nodal or visceral dissemination [17,21]. The histological evaluation combined Patnaik grade II classification with the Kiupel system, which reduces inter-observer variability and correlates better with survival and metastatic potential [18,21]. The biological behavior of canine mast cell tumors is known to vary according to the histological grade, with significant differences described in proliferation markers, c-KIT expression patterns, mitotic activity, and signaling pathways [18,21]. Although mitotic index data were not available for all cases due to variability in routine histopathological reporting, the subset of tumors with available data showed a balanced distribution between low and high categories, consistent with the overall tumor grade profile of the cohort. For this reason, only grade II mast cell tumors were included in the present study in order to reduce the variability related to tumor grade and to allow for a more consistent evaluation of the molecular pathways analyzed. The inclusion of additional histological grades, as well as their interaction with reproductive status, would be of interest for future studies. Nevertheless, the present study has some limitations that should be considered when interpreting the results, particularly the limited number of animals included. However, the distribution of individuals among groups was balanced and, although the study was not designed as a formal case–control study, the selection of animals considered age and breed, and a balanced number of intact and neutralized females were included in both the MCT and control groups to minimize potential confounding effects. Normal skin was used as the reference tissue, as previously reported in studies that employed skin as a control in cancer research [5]. Although it may not represent an ideal control for mast cell tumors due to the greater cellular heterogeneity of normal skin (see below), this approach represents the most feasible methodological alternative for comparative studies. In addition, normal canine skin has been shown to express LHR and FSHR in several cell types [5], supporting its use as a biologically relevant reference for studies evaluating hormonal receptor expression. Therefore, while not a perfect control, normal skin represents the most practical and biologically relevant tissue for comparison in studies of cutaneous mast cell tumors.

c-KIT encodes the Stem Cell Factor (SCF) receptor KIT, essential for mast cell proliferation, survival, and maturation [16]. Constitutive activation—via mutations or altered localization—contributes to the uncontrolled proliferation, apoptosis resistance, and aggressiveness of MCTs [22,23]. In the present study, c-KIT expression was evaluated at the mRNA level by quantitative PCR, and the mutational status of the c-KIT gene was not investigated. Activating mutations in c-KIT have been associated with altered KIT expression, increased proliferation, and more aggressive biological behavior in canine mast cell tumors [22,24]. Since these mutations may influence signaling pathways and gene expression profiles, this aspect should be considered when interpreting the present results. In our study, the dogs with MCTs tended to show higher c-KIT expression than the controls. However, unlike previous findings showing a positive association between KIT and proliferation markers [25,26], we observed low Ki-67 and PCNA expression in tumors, with higher expression in the controls. This finding should be interpreted by considering that normal skin contains physiologically proliferating keratinocytes with high turnover rates [27], while tumor samples comprise a mixed population of neoplastic mast cells, stroma, and inflammatory cells that can affect the overall mRNA levels detected. These results align with reports showing that c-KIT expression by RT-qPCR does not correlate with tumor grade or proliferative index [16,28]. This suggests that KIT signaling may preferentially promote mast cell survival, migration, or maturation rather than active proliferation [26,29]. It should be considered that tumor aggressiveness reflects a balance between proliferation and apoptotic loss; thus, low proliferative markers do not necessarily imply a non-aggressive clinical course [30]. Interestingly, the neutered MCT dogs tended to had greater c-KIT expression than the intact dogs. Although not previously described in MCTs, whether hormonal influences could modulate KIT expression in canine mast cell tumors remains speculative. These data are novel and should be regarded as preliminary, but this differential expression may be associated with the impact of gonadectomy on MCTs, as previously suggested [1]. Regarding the presence of c-KIT transcripts in the skin of control dogs, it is physiologically expected primarily due to the resident mast cells, which are known to express KIT in this species [31]. In contrast to humans, where KIT is expressed in epidermal basal cells and adnexal structures [32], KIT expression in normal canine skin appears more restricted, with no consistent immunoreactivity reported in these cell populations [31]. Therefore, the observed differences between intact and spayed/neutered animals might reflect variations in mast cell density, distribution, or other indirect factors rather than direct hormonal regulation of c-KIT transcription.

Research on IGF-1 in canine MCTs is scarce. Studies on other tumor types have reported variable trends: increased serum IGF-1 in mammary carcinoma [33] and other cancers [34,35], but reduced IGF-1 in hepatic cancer [36] and in thyroid carcinoma [37]. In our study, IGF-1 expression by qPCR is lower in the MCTs than in the controls. The differences between mRNA and protein localization may explain this, since IGF-1 protein can be produced by stromal cells or accumulate in a tumor microenvironment independently of tumoral transcription [38]. Thus, IGF-1 expression in our study suggests that active proliferation in MCTs at this stage does not include this factor and/or that other biological processes, such as mast cell survival, are induced.

VEGF expression resembled IGF-1 expression, as it is lower in the MCTs than in the controls, which contrasts with the expectations for pro-angiogenic tumors [39]. Previous studies have suggested that, although MCTs express VEGF and its receptors, autocrine VEGF signaling is not a major regulator of growth [40,41]. Patruno et al., on the other hand, showed VEGF modulation independent of proliferation, indicating a predominantly paracrine angiogenic role [42]. Overall, VEGF and IGF-1 expression in the dogs with cancer and the control dogs are consistent with Ki-67 and PCNA expression, and suggest that their proliferation in MCTs is not explained by an increase in these transcripts’ concentrations. Indeed, there are several biological mechanisms in cancer that are regulated independently of an increase in mRNA expression of proliferative factors [43].

Interestingly, the neutralized females showed higher VEGF expression than the intact females with MCTs, although they were still lower than in the controls. An ovariectomy induces hormonal and metabolic changes—including altered gonadotropins, weight gain tendencies, and stromal remodeling—that can modify tissue homeostasis, influence inflammatory and angiogenic signaling, and promote a more reactive tumor microenvironment [44]. These changes may enhance local transcription of growth-related genes via compensatory signaling or increased stromal participation [13]. Indeed, this difference in the neutered vs. intact MCT females is consistent with the idea that hormonal alterations due to gonadectomy at an early age could influence the pathophysiology of MCTs [3], and is consistent with the LHR and FSHR expression.

It is well known that LH receptors are expressed in multiple tissues beyond the reproductive system [3]. LHR expression has also been reported in several neoplastic tissues, such as lymphoma [45], hemangiosarcoma [46], and adrenocortical tumors [47]. In MCTs, only one prior study [7] has evaluated the number of cells positive to LHR, and reported higher expression in gonadectomized animals. Although that study was limited in the number of animals and lacked neutered males and a control group, these findings raise the possibility that neutralized females have an increased risk of developing MCTs [1], potentially associated with LHR upregulation. In our study, we included dogs with MCTs and control females who were intact and neutered, which allowed us to examine the roles of gonadectomy and tumor within the same study design. LHR expression was significantly higher in cutaneous MCTs compared with healthy skin, supporting the idea that the presence of this receptor is not incidental but may represent a relevant component of mast cell tumor biology. Regarding FSH, researchers have demonstrated its extragonadal expression in the canine lower urinary tract [4,48] and in the skin [5], supporting the plausibility of finding FSHR in other canine tissues or tumors. In line with this, a recent study evaluated LHR and FSHR expression in canine mammary tumors and identified gonadotropin receptor dysregulation as associated with tumor malignancy and reproductive status [6]. To the best of our knowledge, this was the first study to assess FSHR expression in canine MCTs, revealing its significantly higher expression in dogs with neoplasia, a finding that parallels our results for LHR. Taken together, the expanding evidence for extragonadal gonadotropin receptor expression across multiple tissues and tumor types suggests that FSHR—and more broadly, gonadotropin signaling—could play an underrecognized role in the biology of canine neoplasms. This emerging pattern underscores the need for deeper investigation into how chronic post-gonadectomy hypergonadotropinemia and local receptor regulation might influence tumor initiation, progression, and microenvironmental remodeling.

Finally, it is suggested that sex steroid hormones may play a role in regulating the normal function of skin appendages and in the development of neoplasms; however, the association with MCTs remains controversial. One study reported cytosolic receptors for estrogen and progesterone in canine MCTs [49], while another found that six of nine MCTs did not contain ERα [50]. In addition to the scarce and contradictory evidence regarding steroid hormone receptors in canine MCTs, our findings provide new insight into the potential role of endocrine pathways in MCT biology. We observed a significant reduction in ERα expression in the dogs with MCTs compared with the healthy controls, indicating that ERα downregulation may accompany, or even facilitate, neoplastic transformation. This pattern is biologically relevant given that ERα is normally expressed in multiple cutaneous structures (epidermis, hair follicles, and sebaceous glands) [51]. In the present study, ERα expression was significantly influenced by reproductive status, with a significant group–reproductive status interaction detected by the statistical model, suggesting that the decreased ERα expression observed in the tumor-bearing dogs was likely driven by tumor-associated processes rather than by reproductive status alone [44]. This finding is biologically plausible, as steroid hormone receptor expression is known to be modulated by the endocrine environment in canine tissues. Variations in hormone receptor expression according to tumor histology have been documented in other canine neoplasms, such as perianal gland tumors, where receptor expression correlated with histological grade, although the effect of gonadectomy was not evaluated in that study [52]. However, to our knowledge, the specific effects of gonadectomy on ERα expression in canine mast cell tumors have not been directly investigated. The consistency of our observations with the known biological plasticity of steroid hormone receptor expression in canine tissues supports that the differences detected are meaningful, although the underlying mechanisms remain speculative. Notably, while our findings demonstrate associations between reproductive status and receptor expression, they do not establish a causal effect of gonadectomy on MCT development or progression. Nevertheless, given the relatively small sample size, these results should be interpreted with caution, and further studies with larger cohorts are warranted to better define the biological and clinical relevance of these findings.

In our study, the increased LHR- and FSHR-related transcript levels in tumor tissues—without a corresponding rise in proliferative markers, such as Ki-67, PCNA, VEGF, and IGF-1, and alongside reduced ERα expression—raises the possibility that gonadotropin signaling may preferentially promote cell survival and invasive potential rather than active proliferation. This pattern could reflect activation of pro-survival pathways; upregulation of anti-apoptotic mediators; or the induction of cellular changes associated with enhanced motility, reduced adhesion, and extracellular matrix remodeling that facilitate tissue invasion and dissemination [53]. This interpretation is consistent with previous evidence showing that LH/hCG can suppress apoptosis in LHR-expressing ovarian epithelial and cancer cells, including protection against chemotherapy-induced cell death [54]. An additional possibility is the presence of alternative FSHR/LHR splice variants in tumor tissues, which could redirect signaling toward non-canonical, invasion-associated pathways [53]. Overall, the 5- to 10-fold increased expression of FSH and LH receptors suggests a potential role for their downstream mechanisms in tumorigenesis. Indeed, a recent review reported their relevance through the development of hormone analogues as cancer theragnostic agents [55].

To our knowledge, no previous studies have evaluated the relationship between MCTs, gonadal status, and the expression of LHR, FSHR, and ERα. Therefore, the present results provide novel information that may help to explore the possible role of hormonal receptors in the biology of canine MCTs. Collectively, the downregulation of ERα together with the upregulation of LHR and FSHR in MCTs appears to reflect a broader shift in hormonal receptor signaling in MCTs. Whether these alterations reflect an intrinsic tumor-associated remodeling of endocrine pathways or are influenced by reproductive status remains to be determined. Because steroid and gonadotropin receptors are known to influence processes, such as survival, invasion, angiogenesis, and differentiation, in other tumor types, the combined pattern observed here leaves open the possibility that integrated hormonal signaling changes could be involved in MCT development and biological behavior, although this remains speculative and requires further investigation.

## 5. Conclusions

In conclusion, the presence of a tumor was associated with increased *LH* and *FSH* receptors and *c-KIT* expression, whereas angiogenic factors and proliferative factors (*PCNA*, *Ki-67*, *IGF-1*, *VEGF*, and *ERα*) were lower. Moreover, *c-KIT* and *VEGF* expression in the MCT dogs were greater, but *LHR* mRNA was lower in the neutralized than intact females.

## Data Availability

The data that support the findings of this study are openly available in Figshare at http://doi.org/10.6084/m9.figshare.31037995.

## Supplementary Materials

The following supporting information can be downloaded at: https://www.mdpi.com/article/10.3390/ani16091364/s1, Supplementary Table S1. Breeds and age of the individual dogs included in the study. Supplementary Table S2. Clinicopathological characteristics of dogs with cutaneous mast cell tumors.

## Abbreviations

The following abbreviations are used in this manuscript:

LHR	Luteinizing Hormone Receptor
FSHR	Follicle-Stimulating Hormone Receptor
MCT	Mast Cell Tumor
ERα	Estrogen Receptor Alpha
IGF-1	Insulin-Like Growth Factor
IGF-1R	Insulin-Like Growth Factor Receptor
VEGF	Vascular Endothelial Growth Factor
PCNA	Proliferating Cell Nuclear Antigen
qPCR	Quantitative PCR
SCF	Stem Cell Factor
RT-qPCR	Reverse Transcription Quantitative PCR
LH	Luteinizing Hormone
FSH	Follicle-Stimulating Hormone
hCG	Human Chorionic Gonadotropin
