# Supplementary material for: Gene Expression of Hormone Receptors and Growth Factors in Intact and Neutralized Female Dogs, Both Healthy and with Cutaneous Mast Cell Tumors
Source: Animals (Basel). 2026 Apr 29;16(9):1364. doi: 10.3390/ani16091364 (PMC13162918; doi:10.3390/ani16091364)
Supplement: Supplementary file 1 [file animals-16-01364-s001.zip › Supplementary Table 1.pdf]

Supplementary Table 1. Breeds and age of the individual dogs included in the study.

ID	Group	Age (years)	Breed	Reproductive status
**1**	MCT	11	Poodle	Intact
**2**	MCT	10	Cimarrón Uruguayo	Intact
**3**	MCT	7	Mixed-breed dog	Intact
**4**	MCT	7	Dogo Argentino	Intact
**5**	MCT	6	American Pit Bull Terrier	Intact
**6**	MCT	7	Labrador Retriever	Intact
**7**	MCT	1.5	Mixed-breed dog	Intact
**8**	MCT	8	American Staffordshire Terrier	Intact
**9**	MCT	10	Golden Retriever	Intact
**10**	MCT	13	Labrador Retriever	Spayed
**11**	MCT	5	Mixed-breed dog	Spayed
**12**	MCT	4	Mixed-breed dog	Spayed
**13**	MCT	7	Labrador Retriever	Spayed
**14**	MCT	7	Mixed-breed dog	Spayed
**15**	MCT	9	Dachshund	Spayed
**16**	MCT	10	Golden Retriever	Spayed
**17**	MCT	8	Mixed-breed dog	Spayed
**18**	MCT	11	Labrador Retriever	Spayed

**19**	MCT	9	Rhodesian Ridgeback	Spayed
**20**	CONTROL	2	Mixed-breed dog	Spayed
**21**	CONTROL	6	Mixed-breed dog	Intact
**22**	CONTROL	5	Dalmatian	Intact
**23**	CONTROL	2	American Pit Bull Terrier	Intact
**24**	CONTROL	3	American Pit Bull Terrier	Intact
**25**	CONTROL	1	Labrador Retriever	Intact
**26**	CONTROL	1	Mixed-breed dog	Intact
**27**	CONTROL	1	Labrador Retriever	Intact
**28**	CONTROL	6	Mixed-breed dog	Intact
**29**	CONTROL	1	Labrador Retriever	Intact
**30**	CONTROL	11	Mixed-breed dog	Spayed
**31**	CONTROL	11	Mixed-breed dog	Spayed
**32**	CONTROL	10	Mixed-breed dog	Spayed
**33**	CONTROL	14	Mixed-breed dog	Spayed
**34**	CONTROL	7	Mixed-breed dog	Spayed
**35**	CONTROL	5	Mixed-breed dog	Spayed
**36**	CONTROL	11	Mixed-breed dog	Spayed
**37**	CONTROL	6	Mixed-breed dog	Spayed
**38**	CONTROL	12	Poodle	Spayed
**39**	CONTROL	6	Mastin	Spayed
