# Supplementary material for: Gene Expression of Hormone Receptors and Growth Factors in Intact and Neutralized Female Dogs, Both Healthy and with Cutaneous Mast Cell Tumors
Source: Animals (Basel). 2026 Apr 29;16(9):1364. doi: 10.3390/ani16091364 (PMC13162918; doi:10.3390/ani16091364)
Supplement: Supplementary file 1 [file animals-16-01364-s001.zip › Supplementary Table 2.pdf]

Supplementary Table 2. Clinicopathological characteristics of dogs with cutaneous mast cell tumors

ID	Grade (Kiupel)	Tumor location	Tumor size (cm)
**1**	High	**Forelimb**	2.1 x 2.3
**2**	High	**Hindlimb**	2.0 x 2.0
**3**	High	**Hindlimb**	3.0 x 2.1
**4**	Low	**Thorax**	2.0 x 2.1
**5**	High	**Vulva**	2.0 x 2.3
**6**	Low	**Hindlimb**	8.0 x 10.1
**7**	Low	**Head**	1.0 x 1.0
**8**	High	**Vulva**	5.5 x 3.1
**9**	Low	**Thorax**	4.0 x 3.7
**10**	Low	**Thorax**	3.0 x 3.0
**11**	High	**Forelimb**	3.0 x 2.1
**12**	Low	**Thorax**	5.3 x 5.0
**13**	Low	**Thorax**	2.1 x 1.0
**14**	High	**Hindlimb**	4.5 x 3.2
**15**	Low	**Head**	1.5 x 1.6
**16**	Low	**Head**	2.0 x 2.0
**17**	High	**Forelimb**	10.1 x 10.5
**18**	High	**Thorax**	8.0 x 7.4

**19**	High	**Thorax**	2.2 x 1.9
**20**	High	**Forelimb**	2.0 x 2.2
